# The cost-effectiveness of inventory reserves for preventing drug shortages in Germany: a health-economic evaluation

**DOI:** 10.1038/s41598-026-53010-8

**Published:** 2026-06-08

**Authors:** Afschin Gandjour

**Affiliations:** Frankfurt School of Finance & Management, Adickesallee 32-34, 60322 Frankfurt am Main, Germany

**Keywords:** Drug shortages, Germany, Inventory reserves, Cost-effectiveness, Health care, Mathematics and computing, Medical research

## Abstract

Based on a recent legislative change in Germany, maintaining a six-month inventory reserve for pharmaceuticals has become the primary strategy used by the German government to prevent shortages and stockouts. This study aimed to estimate the number of drug shortages preventable under the medicines stockpiling mandate and to determine the maximum stockpiling duration that remains cost-effective. The study adopted the perspective of the German social health insurance and analyzed a publicly available database containing information on all past drug shortages in Germany from the first entry in 2012 to May 2023 (n = 2768). The proportion of preventable drug shortages was estimated as a function of inventory duration. Additionally, secondary data were used to ascertain the costs associated with inventory carrying (including storage, capital, and expiration costs) and the gain in quality-adjusted life years (QALYs) resulting from avoiding relevant shortages of medication packages. Maintaining a six-month inventory could prevent approximately 70% of drug shortages. The empirical analysis revealed a diminishing incremental benefit of extending the drug supply duration, leading to an increasing marginal cost-per-QALY ratio. Based on a health opportunity cost–based cost-effectiveness threshold, the cost-effective upper limit of inventory duration under the current assumptions was approximately 922 days. This estimate was particularly sensitive to assumptions about FIFO (“first in, first out”) warehouse procedures, number of drug shortages, and the valuation of health gains. This study indicates that Germany’s recently introduced six-month inventory mandate could substantially reduce drug shortages and deliver good value for money, with a very high probability of cost-effectiveness across the willingness-to-pay thresholds examined in the sensitivity analysis. However, key uncertainties—especially around FIFO procedure compliance, health opportunity costs, and the clinical consequences of shortages—warrant ongoing monitoring and periodic reassessment as new evidence becomes available.

**Supplementary Information:**

The online version contains supplementary material available at 10.1038/s41598-026-53010-8.

## Introduction

In 2023, medicine shortages continued to affect every surveyed European country, and in the majority (65%), the situation was reported to have worsened or remained as severe as in the previous year^1^. The specific reasons for drug shortages can vary depending on the healthcare system, regulatory environment, and market dynamics of each country. However, some common themes are observed globally. These include problems in the manufacturing process, such as quality control issues or production disruptions; regulatory requirements and inspections; disruptions in the global supply chain, including transportation issues, raw material shortages, or disruptions in the availability of active pharmaceutical ingredients (APIs); market factors, like pricing pressures, reimbursement policies, and profitability concerns; increased demand; and changes in regulations, reimbursement policies, or pricing schemes. An empirical analysis based on German data from 2017 to 2019 found that the probability of a drug shortage increased with demand volatility and decreased with market concentration^2^.

To address drug supply bottlenecks, the German Federal Cabinet approved a bill on April 5, 2023, which included multiple regulatory measures: the relaxation of price rules for pediatric drugs, elimination of reference prices, and adjustments to rebate contract requirements. One provision in the draft legislation proposed a three-month stockpiling requirement for discounted, off-patent drugs. However, this provision was not adopted into law, as the three-month stockpiling obligation was removed prior to parliamentary approval. The law ultimately passed by the German parliament on June 23, 2023, established a six-month stockpiling obligation for rebated, off-patent drugs^3^. Stockpiling, unlike the other measures, affects all drugs except non-rebated ones and was identified as the key lever in preventing drug shortages^4,5^.

Of particular concern are shortages classified as “clinically relevant” (*versorgungsrelevant*) by the Federal Institute for Drugs and Medical Devices (BfArM). This designation is based on case-by-case expert assessment, considering the absence of clinically adequate therapeutic alternatives and the expected deterioration in patient prognosis due to non-availability^6^.

This study aimed to predict how many clinically relevant supply bottlenecks can be prevented by the medicines stockpiling obligation, the cost of implementing the stockpiling obligation, and the maximum cost-effective stockpiling period.

## Literature review on pharmaceutical inventory management

Pharmaceutical inventory management is essential, especially in healthcare settings where the timely availability of medications is critical for patient care. Several studies have explored different approaches to optimize inventory management to mitigate drug shortages and associated costs.

Saedi et al.^7^ present a stochastic model aimed at optimizing inventory policies in healthcare facilities. Their model is designed to minimize total costs, including those incurred from health losses due to drug shortages, although the study does not specify how health losses were measured. The authors highlight the practical benefits of inventory pooling and continuous inventory review policies. By pooling inventories, healthcare facilities can reduce the risk of stockouts and ensure a higher service level, thereby mitigating the impact of supply disruptions on patient care. This study underscores the importance of strategic inventory management in enhancing the reliability of pharmaceutical supply chains.

Buschiazzo et al.^8^ contribute to this field with a simulation model that aims to minimize costs at healthcare facilities. Their analysis includes the costs of maintaining safety stock, stockouts, and supply expiry. While comprehensive, the study does not explicitly address health losses due to drug shortages. The simulation approach allows for the evaluation of different inventory policies under various scenarios, providing valuable insights into cost-effective inventory management strategies. The authors emphasize the need for robust systems that can adapt to fluctuating demand and supply conditions.

In addition to these studies, Uthayakumar and Priyan^9^ explore optimization strategies for pharmaceutical supply chains involving both pharmaceutical companies and hospitals. Their model integrates a continuous-review inventory system, in which stock levels are monitored continuously and replenishment decisions are triggered when inventory falls below a predefined level, together with production and distribution considerations. This study highlights the differences in inventory management priorities between pharmaceutical companies and hospitals, with the former focusing on cost minimization and the latter on ensuring high service levels to meet patient needs.

Salehi et al. ^10^ examine inventory management under supply chain disruptions, proposing an EOQ (Economic Order Quantity) model that accounts for random supply failures. Their research indicates that incorporating the possibility of supply disruptions into inventory decisions, together with strategies such as partial backordering, can mitigate risks associated with supply uncertainties. This approach, while potentially increasing holding costs, is particularly relevant in the context of global supply chain disruptions, such as those caused by natural disasters or geopolitical events.

To investigate the impact of national drug shortages on healthcare delivery, Fox et al.^11^ highlight the significant time and resources medical staff devote to managing these shortages. Their findings indicate that proactive inventory management, including the identification of critical drugs and securing alternative supply sources, is essential for maintaining continuous patient care. The economic and clinical effects of drug shortages are substantial, with the financial impact estimated to be hundreds of millions of dollars annually for health systems across the United States, and clinical impacts including delayed treatment and patient harm.

A study by Ahmadi et al.^12^ introduces intelligent inventory management (IIM) approaches using reinforcement learning techniques (Q-learning and Deep Q-network) to manage perishable pharmaceutical products in a healthcare supply chain. The IIM approaches developed offer near-optimal inventory policies that effectively reduce the risk of product shortages and expirations, while also minimizing total inventory costs compared to traditional periodic review policies. The computational results highlight the superiority of IIM policies in achieving lower total costs, improved service levels for patients, and efficient utilization of storage space in the healthcare system.

Collectively, these studies provide a robust framework for understanding and improving inventory management in healthcare facilities. They highlight the need for models that not only minimize costs but also ensure high service levels to meet patient needs.

## Methods

### Analysis of drug shortages

To examine the number and duration of drug shortages, we utilized the drug shortages database maintained by BfArM in Germany. The BfArM database is essential for monitoring and managing drug supply issues across Germany by tracking and publicly reporting on shortages. It includes all types of medicinal products approved and marketed within the country, including prescription medications, over-the-counter drugs, and specialized pharmaceuticals used in hospitals. This comprehensive database covers various therapeutic areas, providing a complete view of the national supply chain’s status. Data have been collected since 2012.

The database contains detailed information about each drug shortage, including the drug’s commercial name, active ingredients, manufacturer information, and the expected start and end dates of the shortage. It also includes the reasons for the shortage, such as production issues, as provided by the manufacturer. Each entry is marked with the date it was reported to or by the BfArM.

The database is regularly updated in real-time as pharmaceutical manufacturers and other stakeholders report anticipated or actual shortages. The BfArM ensures the database’s accuracy and relevance through regular updates and audits. It is publicly accessible via BfArM’s official website, where users can search for information by drug name, active ingredient, or manufacturer.

According to the BfArM, a supply bottleneck is defined as an interruption in delivery within the usual scope that is expected to last longer than two weeks or a significantly increased demand that cannot be adequately met^13–15^. Even supply shortages lasting less than one day were considered relevant for our analysis, as short-term supply interruptions can accumulate over time and cause significant problems. For instance, repeated short-term bottlenecks can lead to interruptions in the production chain. Moreover, many companies operate on a just-in-time principle, where materials and components are required precisely at the time of processing or assembly. A supply bottleneck of just a few hours can disrupt the production process and delay deliveries to customers. While the database includes all reported shortages, the cost-effectiveness model described below focuses on the incremental impact of extending the inventory reserve. Therefore, shortages lasting only one day have minimal influence on the results, as they are already fully addressed by short reserve durations and do not affect the incremental analysis at longer durations (e.g., 60 vs. 50 days). Furthermore, the shortage probabilities and durations derived from the BfArM database already reflect a supply system with existing inventories at manufacturers, wholesalers, and pharmacies; the model therefore estimates the additional effect of extending reserve levels beyond this baseline.

We included all drug shortages for which a “deletion notice” (“Löschmeldung”) was reported on May 11, 2023. This encompassed shortages that were projected to conclude after that date. Accordingly, the observation period spanned from the first entry in 2012 to May 2023 (n = 2768), including the COVID-19 pandemic years. Pandemic-period observations were not excluded from the main empirical analysis; instead, their potential influence was explored in sensitivity analyses.

In this study, shortage events for the same active ingredient but involving different products (e.g., different manufacturers, pack sizes, or formulations) were aggregated by summation—that is, they were added together, rather than being counted as a single event. This approach follows the public reporting practices of the Federal Institute for Drugs and Medical Devices (BfArM) and reflects how drug shortage figures are typically presented in media reports. However, a shortage of a specific product does not necessarily imply a complete absence of pharmacological treatment, as other presentations of the same active ingredient may remain available and, in some cases, physicians may prescribe a clinically similar substitute. To account for this, only the proportion of shortages classified as “clinically relevant” was incorporated into the cost-effectiveness analysis (CEA). These are shortages with clinically meaningful consequences due to the lack of therapeutic alternatives.

### Cost analysis

The study adopts the perspective of the German social health insurance (SHI) and includes various stockpiling-related costs for pharmaceuticals, such as storage, capital, and expiration. The storage costs considered reflect total storage expenses, including both base rent and operating costs—such as maintaining optimal storage temperatures, systems to monitor storage conditions, security of stored pharmaceuticals, compliance with regulatory standards, inventory management (including software and labor), insurance against damage or theft, utilities, facility maintenance, documentation, and qualified personnel.

The cost of warehouse expansion (e.g., construction and land acquisition) was excluded to avoid double counting. Storage space was valued using commercial rent rates, which represent the cost of occupying warehouse capacity rather than building new capacity. Including both rent-based storage costs and separate construction or land aquisition cost would overstate the true economic cost of stockpiling.

In addition, stockpiling requires manufacturers to produce and hold larger quantities of pharmaceuticals. This ties up capital that could otherwise be used for investments in research, development, or manufacturing. Furthermore, because pharmaceuticals have limited shelf lives, extended stockpiling increases the risk of expiration before products can be dispensed or sold, potentially resulting in financial losses to the manufacturer.

As generic drug prices are regulated in Germany, it can be difficult for drug manufacturers to pass on increased inventory costs directly to sickness funds. This study conservatively assumes (in the sense of higher sickness fund costs) that manufacturers will be able to negotiate special terms or discounts with sickness funds that take into account their inventory costs. Nevertheless, manufacturers may adopt alternative strategies to offset or minimize inventory costs. These include efficient warehousing and effective inventory management to avoid overstocking and reduce storage expenses. In addition, geographically diversified production can enhance supply-chain resilience and flexibility and thereby reduce the need for large inventory buffers.

### Storage costs

Pharmaceutical storage costs vary depending on location, service provider, and technical requirements. Estimates are typically calculated per square meter per month and include both base rent and operating expenses—such as staff, energy, equipment, temperature control, and regulatory compliance. A plausible all-inclusive range is €5–20 per square meter per month. While €10.50/m^2^/month represents the upper end of base rent in high-cost areas such as Munich^16^, this figure excludes pharmaceutical-specific operational and compliance-related expenses. Therefore, the €5–20/m^2^/month range remains appropriate as a comprehensive estimate for pharmaceutical storage, accommodating both regional variation and differences in storage requirements—particularly the higher costs associated with cold chain storage compared to ambient conditions.

The proportion of medicines requiring refrigerated storage has grown due to the increasing share of biopharmaceuticals, vaccines, and other temperature-sensitive products. While transport data suggest that 17–31% of pharmaceutical shipments involve cold chain logistics ^17^, this figure is inflated by bulk volume. For warehouse planning purposes, a more accurate metric is the share of distinct pharmaceutical products (SKUs) requiring continuous refrigeration, which is estimated at 10% to 20% based on regulatory and formulary data.

To convert area-based storage costs into per-pack costs, we apply an average storage density of 1000–1200 finished-dose packs per square meter and factor in a cold-chain share of 10–20%. This yields an expected cost of approximately €0.005–0.02 per pack per month. For triangulation, pallet-based pricing supports this estimate: typical cold-chain storage fees in Western Europe range from €25 to €40 per pallet per month (e.g., ^18^), while ambient storage ranges from €15 to €25. Given a standard pallet load of 1500–3000 packs, this also translates to approximately €0.004–0.02 per pack per month.

In the base-case analysis, we therefore assume an average warehousing cost of €0.01 per pack per month and vary this parameter between €0.005 and €0.03 per pack per month in sensitivity analyses to reflect uncertainty in storage density, regional cost variation, cold-chain share, and partial pallet utilization.

### Cost of lost durability

The cost of lost durability should naturally be minimized by ensuring that pharmaceuticals do not reach their expiration date before being sold to the end consumer. However, various factors may still lead to this situation, even if drugs are only stored at the manufacturer for a specified time. Sometimes manufacturers might produce more than needed based on forecasts or other economic considerations, leading to excess inventory. Delays or issues in the supply chain can prevent products from arriving at pharmacies or consumers on time. Additionally, new products or changes in treatment guidelines can reduce the demand for older products. If manufacturers anticipate longer storage times, they may need to modify formulations or packaging to preserve shelf life, but such changes can be technically, economically, and regulatorily challenging. These challenges may arise from regulatory requirements, packaging or fomulation changes, or other product-specific constraints. In addition, some products are subject to seasonal demand fluctuations, which may lead to faster sales during certain periods than others.

To address these issues, stock must be actively rotated so that batches with the earliest expiry dates are used first. In pharmaceutical logistics, this is ideally done according to the FEFO (“first expired, first out”) principle, under which products are issued in order of their expiry date rather than simply in the order in which they entered storage. By contrast, FIFO (“first in, first out”) means that the oldest stock in storage is used first, regardless of its exact expiry date. FIFO may approximate FEFO when products have similar shelf lives, but FEFO is the more appropriate approach for pharmaceuticals because it directly minimizes the risk of expiry during storage. Such stock-rotation systems are standard among generic pharmaceutical manufacturers and help reduce wastage by ensuring timely use of batches before expiration.

Typically, the shelf life of most pharmaceuticals is between two and three years. In the base case analysis, we assume that generic manufacturers implement FIFO/FEFO with a very high, but not perfect, degree of adherence, corresponding to approximately 90–95% effective FIFO adherence. For example, if the inventory period is six months and the expiration date is 2.5 years, then under 100% FIFO adherence the products would still have two years of remaining shelf life at the point of sale. This remaining period is generally sufficient to ensure that medicines can be distributed, dispensed, and used well within their effective shelf life.

Nevertheless, a stockpile—whether it corresponds to three or six times monthly consumption—must be managed with continuous turnover. Even under ongoing replacement, a fraction of the inventory will expire due to imperfect FIFO adherence, unforeseen demand changes, or supply-chain disruptions. Therefore, the cost of lost durability remains relevant even under well-functioning inventory management systems and is explicitly included in the analysis. In the sensitivity analysis, drug durability is assumed to decline linearly over time, thereby capturing the time-dependent risk of expiration.

### Cost of capital

The Weighted Average Cost of Capital (WACC) for generic manufacturers can vary depending on several factors, including company-specific risk profiles, financial structure, and access to capital markets. It is important to note that there is no single rate applicable to all generic manufacturers. However, generic manufacturers typically have lower capital costs than innovative pharmaceutical companies, as they face lower R&D expenditures and operate in broader, more predictable markets.

In this study, the cost of capital is used to represent the opportunity cost of tying up financial resources in inventory reserves—that is, the foregone return on funds that must be used to produce and store pharmaceuticals instead of being allocated to other productive uses, such as investment or debt reduction. The opportunity cost reflects the return (or interest) not earned on the capital bound in inventory.

Based on industry benchmarks, the WACC for generic manufacturers is generally estimated to lie between 8 and 12%. We use a 10% WACC in the base case, and vary this between 8 and 12% in sensitivity analysis to reflect plausible variation in financial conditions across manufacturers.

### Cost-effectiveness analysis

We were interested in the number of rebated generic packages affected by clinically relevant shortages, because the stockpiling mandate applies specifically to generics under rebate contracts with SHI funds. To estimate the number of clinically relevant rebated packages, the annual volume of rebated generic packages sold in Germany $\left(N\right)$ was multiplied by the estimated share of generic drugs affected by clinically relevant shortages. This share was derived from the number of observed shortage events $\left(D\right)$, restricted to the share classified as clinically relevant $\left(S\right)$, the number of generics with rebate contracts $G$, and the proportional volume of rebated generics $V$. Thus

1 $$U=\frac{N\cdot S\cdot D}{G/V}.$$

In 2023, the total number of generic drugs on the German market was estimated at approximately 29,000. This estimate was extrapolated from the 21,339 generic drugs with rebate contracts between manufacturers and sickness funds, using the proportional volume of rebated generics (73.8%) ^19,20^. The number of rebated drug packages sold in Germany $\left(N\right)$ was 383 million in 2021^21,22^.

It is noteworthy that there is substantial year-to-year variation in clinically relevant drug shortages, ranging from 56 in 2023 to 426 in 2020^23^. On average, BfArM classifies about 44% of all reported shortages as clinically relevant. Analyses of the BfArM shortage lists for 2022/2023 show that only a relatively small number of substances are clearly still patent-protected (e.g. semaglutid, dulaglutid, insulin degludec, ertugliflozin, encorafenib, bosutinib, sodium zirconium cyclosilicate and a few modern biologics/oncology agents), so that about 95% of shortage-affected active substances are off-patent generics. This implies that the clinically relevant share for the generics segment must lie in a relatively narrow interval of roughly 41–46% and thus cannot deviate strongly from the overall 44% figure. Excluding the pandemic period (2020–2022), which caused atypical supply chain interruptions, the observed percentage of clinically relevant shortages is 38%, so we use 38–46% as the range for sensitivity analyses.

Despite this variation in clinically relevant drug shortages, the total number of reported drug shortages has remained stable around 500 over the past 12 months. For the base case analysis, this value was used, and in the sensitivity analysis, the value was varied between 400 and 600. The base-case values and ranges for key model inputs used in the cost-effectiveness and sensitivity analyses are summarized in Table 1.

**Table 1:** Input values and distributions used in the base case cost-effectiveness analysis and sensitivity analysis. All costs are in Euros.

Input parameter	Base-case value	Distribution for PSA	Shape parameters (notation)	Reference(s)
Storage cost (cents/pack/month)	1	Triangular	*a* = 0.5 (min), *m* = 1 (mode), *b* = 3 (max)	van Berkel^17^, Waredock^16^, Zendeq^18^
FIFO adherence (%)	92.5	Beta	*α* = 46, *β* = 4 (mean ≈ 0.925, precision ≈ 50)	Assumption (bounds reflect best-practice and complete failure)
Number of active drug shortages (point-in-time)	500	Triangular	*a* = 400, *m* = 500, *b* = 600	BfArM database
MCID for HRQoL loss per day	0.085	Triangular	*a* = 0.07, *m* = 0.085, *b* = 0.10	IQWiG^24^
Proportion clinically relevant shortages	0.44	Triangular	*a* = 0.38, *m* = 0.44, *b* = 0.46	BfArM^23^
Weighted-average cost of capital (%)	10	Triangular	*a* = 8, *m* = 10, *b* = 12	Damodaran^25^

PSA, probabilistic sensitivity analysis; FIFO, first-in, first-Out; MCID, minimum clinically important difference; HRQoL, health-related quality of life.

Quality-adjusted life years (QALYs) are used to measure health outcomes by combining the quantity and quality of life lived, providing a standard metric in health economics to assess the effectiveness and cost-effectiveness of medical interventions. QALYs are an appropriate measure of health benefit, as they serve as a common currency of health deterioration across therapeutic fields.

An annual time horizon was used because key model inputs, such as package volumes, were available primarily as yearly aggregates, because a one-year period captures variation in drug shortages over the course of the year, and because the stockpiling mandate represents a recurring inventory requirement whose costs and effects can be meaningfully compared in annual terms.

In this context, the incremental number of avoided clinically relevant package shortages was calculated for each incremental increase in inventory duration. The incremental cost was then divided by the incremental number of avoided clinically relevant package shortages, resulting in the incremental cost per avoided package shortage. To estimate the corresponding QALY gain, we first derived the average number of defined daily doses (DDDs) per rebated generic package by multiplying the total prescribed generic DDDs in Germany (27.47 billion) by the share of rebated generics and dividing the result by the number of rebated generic packages. This value was used to convert each avoided package shortage into an estimated number of treatment days preserved. The QALY gain per avoided package shortage was then assumed to equal the QALY loss avoided by maintaining access to the clinically relevant drug. This daily QALY loss was determined on the basis of the BfArM definition of clinically relevant health loss, namely deterioration in patient prognosis due to the non-availability of a drug. The clinically relevant health loss was equated to the minimum clinically important health loss (minimum clinically important difference, MCID) in the absence of the drug. Thus, a clinically relevant health loss was considered to result in a clinically meaningful reduction in health. We used the MCID from the EQ-5D visual analogue scale (VAS), as it is a validated tool for measuring health-related quality of life^24^. The midvalue of the MCID range (0.085) was selected from the validated range of 0.07 to 0.10 for the EQ-5D VAS. However, the VAS is a rating scale rather than a preference-based utility measure. In the absence of a generic utility-based estimate of health loss applicable across therapeutic areas, the VAS-based MCID was therefore used as a pragmatic proxy for clinically meaningful health loss. Under this assumption, the selected MCID was converted into a daily QALY loss by dividing it by 365. The limits of the MCID range were varied in sensitivity analyses to assess the robustness of the findings.

While the MCID can be disease-specific, an assumed average value is appropriate for our analysis, as the cost-effectiveness model is based on an average across therapeutic areas. This is consistent with the fact that the mandated six-month stockpiling period does not vary by therapeutic area.

The model assumes a linear relationship between shortage duration and QALY loss due to the absence of detailed clinical progression data across therapeutic areas—that is, each day without a clinically relevant drug contributes equally to the overall loss of health-related quality of life. We acknowledge that the actual relationship may differ by condition. In some cases, health loss may be convex—where deterioration accelerates with the length of the interruption in therapy, such as in progressive diseases (e.g., cancer, epilepsy). In others, it may be concave, with a sharp initial decline followed by a plateau due to persistent therapeutic effects or compensatory mechanisms (e.g., behavioral adaptation or secondary treatments). However, because the cost-effectiveness analysis is based on incremental comparisons—e.g., evaluating the additional QALY gains from increasing stockpiling from 50 to 60 days—the shape of the QALY loss function over time is less critical than the local slope. As long as QALY loss increases monotonically with shortage duration, the linear approximation can provide a pragmatic and analytically stable approach across conditions. Moreover, varying the MCID in sensitivity analysis helps capture the uncertainty surrounding both the magnitude and the time profile of QALY losses.

The mathematical model used to calculate the maximum cost-effective storage period is detailed in the Online Appendix. The model extends the storage period to the point at which the marginal cost per QALY gained equals the cost-per-QALY threshold, thereby identifying the maximum storage duration that remains cost-effective under the assumed threshold. The cost-per-QALY threshold $\lambda$ was set at €88,000 in the base case, based on health opportunity costs within the German healthcare system, which operates under budgetary constraints—particularly with respect to tax funding ^26^. If the cost per QALY of an intervention equals this threshold, it indicates that the health benefits provided by the intervention are precisely balanced by the health benefits it displaces elsewhere in the system. Given the absence of a formally accepted threshold in Germany, $\lambda$ was varied in a sensitivity analysis.

The approach of determining the maximum cost-effective storage period based on a cost-per-QALY threshold is conceptually similar to inventory models that optimize stock levels by balancing marginal costs and benefits. In this paper, the storage period is extended until the ICER equals the cost-per-QALY threshold, analogous to models that increase inventory up to the point where the marginal cost of holding an additional unit of stock equals the marginal benefit of reducing stockout risk. However, whereas the present analysis expresses benefits in terms of health gains valued against a cost-per-QALY threshold, traditional inventory models typically define benefits in terms of stockout cost metrics (e.g., ^7^). Full equivalence between the two approaches would require stockout costs to be translated into a health-economic framework, for example by expressing them in terms of QALY losses or their monetary equivalent.

### Sensitivity analysis

To assess the robustness of results, both deterministic and probabilistic sensitivity analyses were conducted. One-way sensitivity analysis was illustrated using a tornado diagram, which ranks the influence of individual input parameters on the net monetary benefit. For each parameter, plausible ranges were informed by published literature or expert assumptions and are detailed in Table 1. The maximum cost-effective supply duration was derived for varying willingness-to-pay (WTP) thresholds by identifying the point at which the ICER equals the WTP. Probabilistic sensitivity analysis (PSA) was performed using Monte Carlo simulation with 1000 iterations, drawing from appropriate probability distributions for all input parameters. Uncertain model parameters were assigned probability distributions according to their scale and the type of information available. We used Beta distributions for parameters bounded between 0 and 1 (e.g. probabilities, proportions) and triangular distributions when only minimum, maximum and most likely values were available from expert opinion or heterogeneous data sources.

## Results

### Analysis of drug shortages

**Fig. 1:**
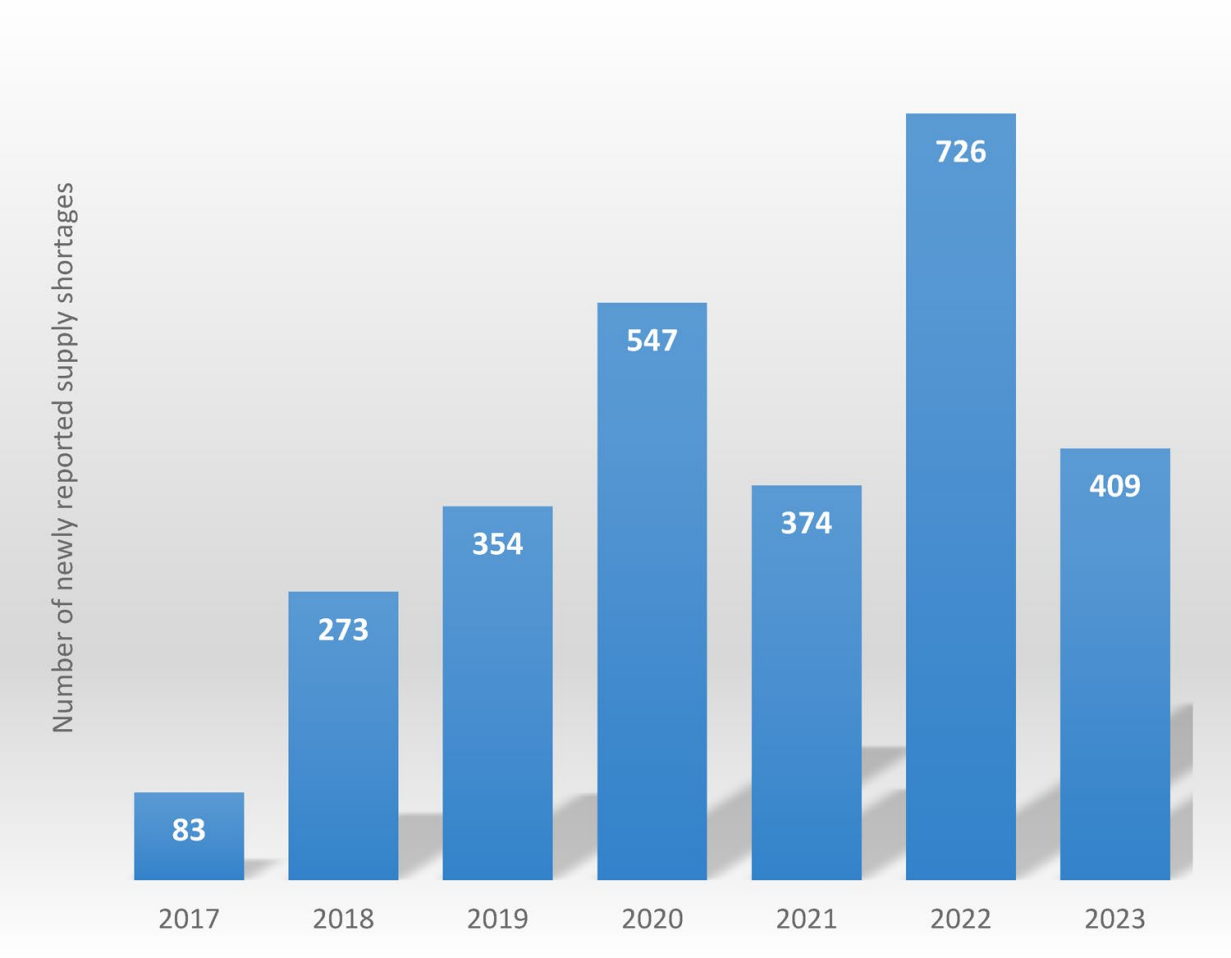
Number of newly reported supply shortages in the database for drug shortages maintained by the Federal Institute for Drugs and Medical Devices (BfArM, Bundesinstitut für Arzneimittel und Medizinprodukte). Data for 2023 cover January to May only.

The number of newly reported drug supply shortages each year between 2017 and May 2023 is shown in Fig. 1. With the exception of 2021, there has been a general increase over time. The anomaly in 2021 could be attributed to temporary stabilization as the world adapted to the pandemic, with specific measures taken to address the shortages that became apparent in 2020. Additionally, increased production capacity for certain drugs to meet the immediate needs of the pandemic may have led to a temporary respite in shortages. However, as the situation evolved and new challenges emerged, the trend of increasing shortages resumed in subsequent years.

**Fig. 2:**
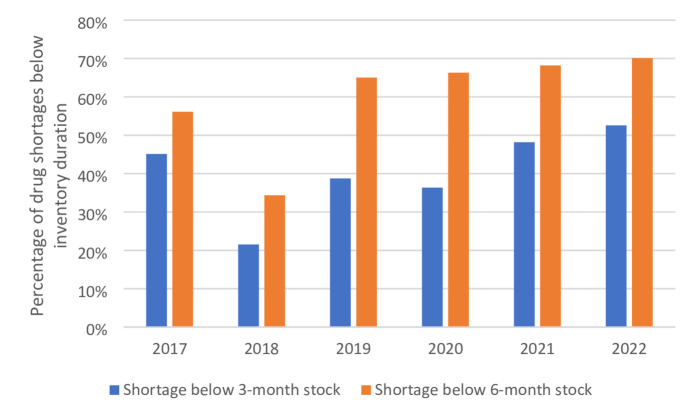
Percentage of drug shortages with durations of less than three months and less than six months.

The median duration of drug shortages was found to be 90 days over the period from 2012 to May 2023. Due to the right-skewed distribution of drug shortages, the mean duration was 195 days. The percentage of drug shortages below an inventory duration of three and six months has varied since 2017, with an increase since 2020, suggesting a higher share of shorter shortages (Fig. 2). With the recent six-month inventory mandate, approximately 70% of drug shortages could be prevented.

**Fig. 3:**
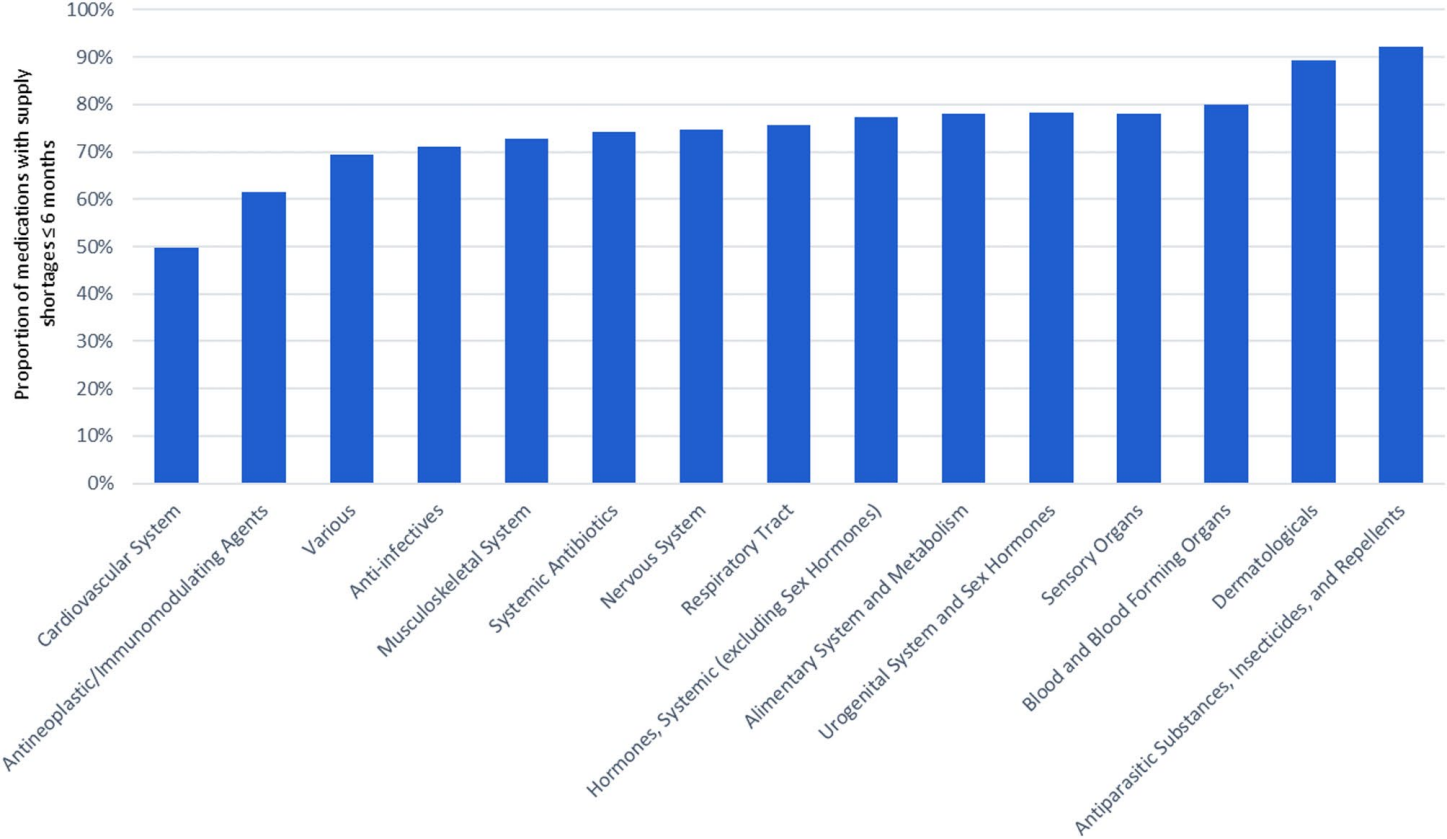
Proportion of medications with supply shortages lasting up to six months by therapeutic category.

Figure 3 shows the proportion of medications with supply shortages lasting six months or less across therapeutic categories. The categories “Dermatologicals” and “Antiparasitic substances, insecticides, and repellents” show the highest proportions, suggesting that a larger share of shortages in these categories could in principle be covered by a mandatory six-month inventory. In contrast, the “Cardiovascular System” category shows a substantially lower proportion, at around 40%, suggesting that shortages in this category would be less likely to be fully mitigated by a six-month inventory.

**Fig. 4:**
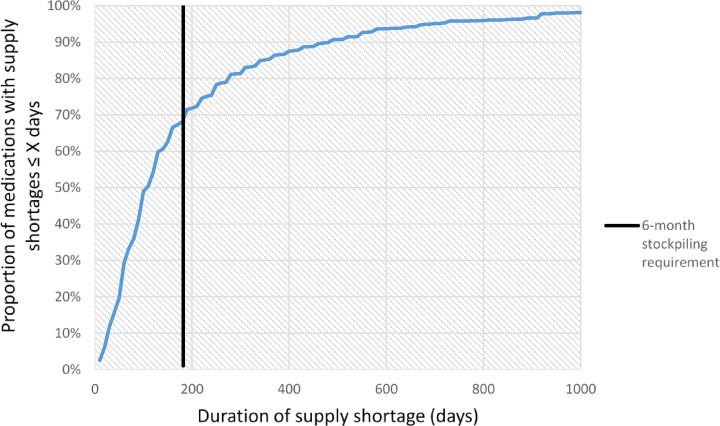
Cumulative distribution of medication supply shortage durations.

As shown in Fig. 4, the cumulative percentage of supply shortages rises steeply at first, indicating a high proportion of supply shortages are resolved within a relatively short duration. The curve begins to plateau around 200 days, suggesting that fewer shortages extend beyond this point.

**Fig. 5:**
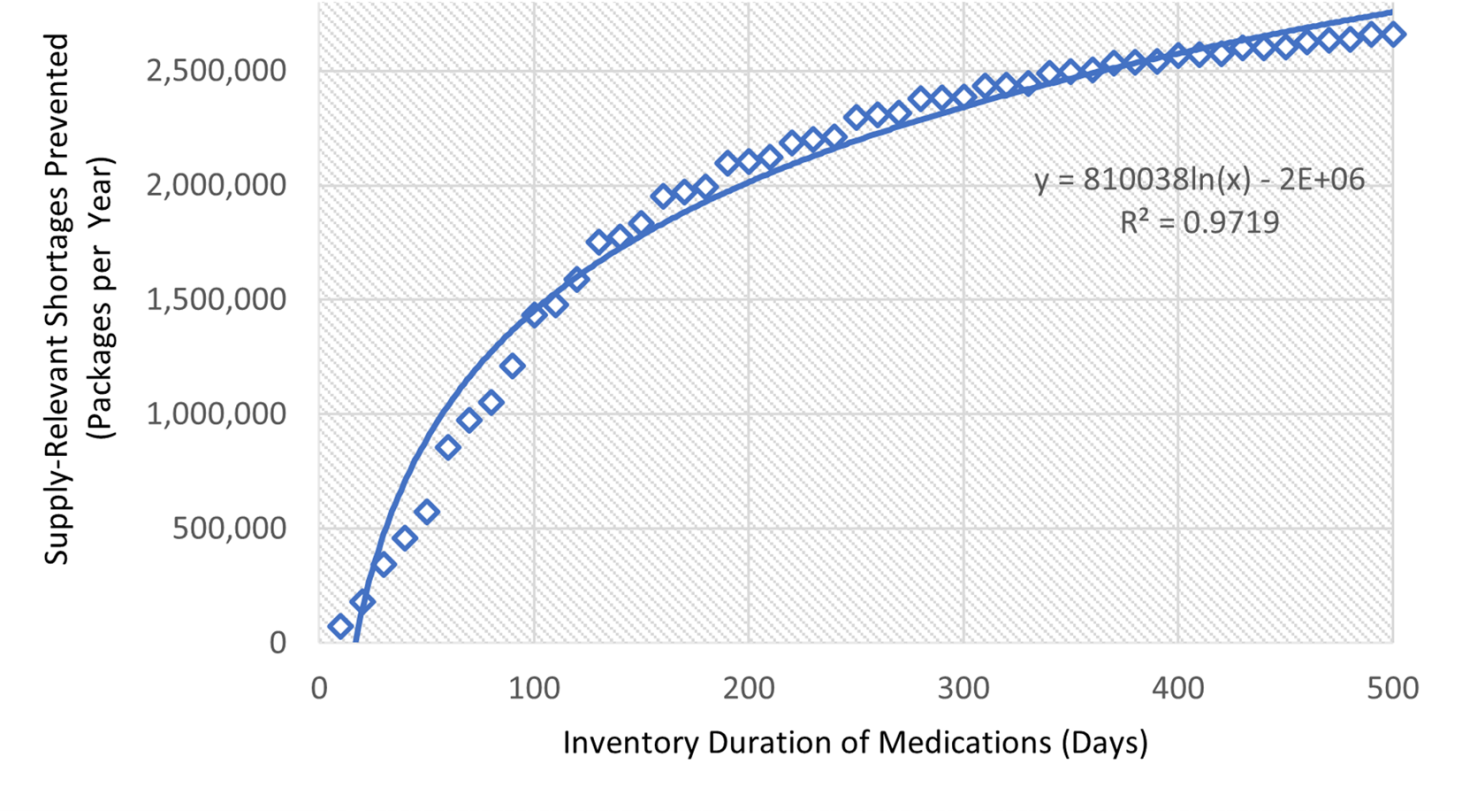
Predicted annual prevention of clinically relevant drug package shortages by inventory duration.

Figure 5 shows the relationship between inventory duration in days and the number of clinically relevant drug shortages prevented per year. The data points were fitted with a logarithmic regression curve based on 50 observations. The regression showed a high coefficient of determination $\left({R}^{2}=0.97\right)$, indicating a strong overall fit. However, the residual plot revealed some systematic deviations, suggesting that the logarithmic curve should be regarded as an empirical approximation rather than an exact representation of the underlying relationship. The graph indicates that longer inventory duration is associated with more prevented shortages, although the incremental benefit decreases as inventory duration increases.

### Cost-effectiveness analysis

Table 2 outlines the projected expenses associated with maintaining a six-month inventory of pharmaceuticals. The main cost driver is the cost of capital. Based on Online Appendix Eq. (9), the maximum storage period for medications was determined to be 922 days, given a threshold willingness to pay of €88,000 per QALY gained. Using Online Appendix Eq. (11), the ICER at a six-month storage period was estimated at €17,361 per QALY gained.

**Table 2:** Estimated annual costs for six-month drug stockpiling.

Cost Item	Amount (€)
Storage costs	22,980,000
Capital costs	107,500,000
Expiration-related costs	32,161,644
Total annual stockpiling costs	162,641,644

### Sensitivity analysis

**Fig. 6:**
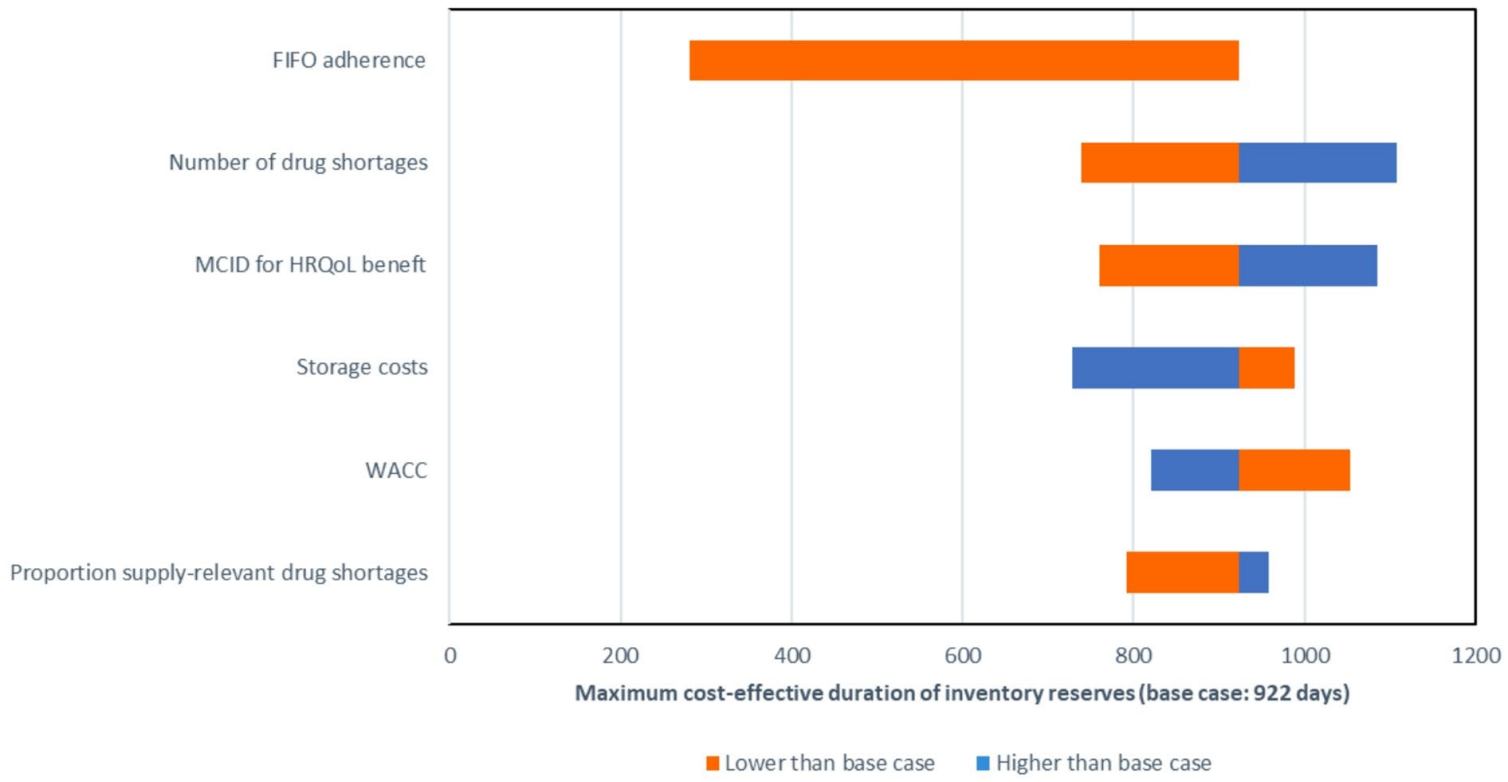
One-way sensitivity analysis on the maximum cost-effective duration of inventory reserves, based on a willingness to pay of €88,000 per quality-adjusted life year. Variables are ordered according to their impact on the result. FIFO, First-In, First-Out; MCID, minimum clinically important difference; HRQoL, health-related quality of life; WACC, Weighted Average Cost of Capital.

The one-way sensitivity analysis indicated that the variable with the greatest impact on the maximum cost-effective duration of inventory reserves was FIFO adherence (Fig. 6). In the sensitivity analysis the maximum cost-effective duration consistently remains above 280 days.

**Fig. 7:**
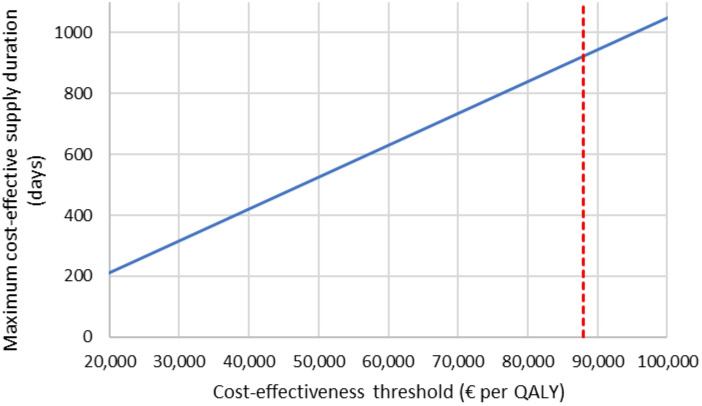
Maximum cost-effective drug supply duration as a function of the cost-effectiveness threshold. The dashed red line indicates the base-case willingness-to-pay (WTP) threshold of €88,000 per QALY gained, representing the opportunity cost benchmark used to determine cost-effectiveness. QALY, quality-adjusted life year.

As shown in Fig. 7, the maximum cost-effective stockpiling period increased monotonically with the WTP threshold, reflecting the higher value placed on avoiding health loss as the opportunity cost benchmark rises.

**Fig. 8:**
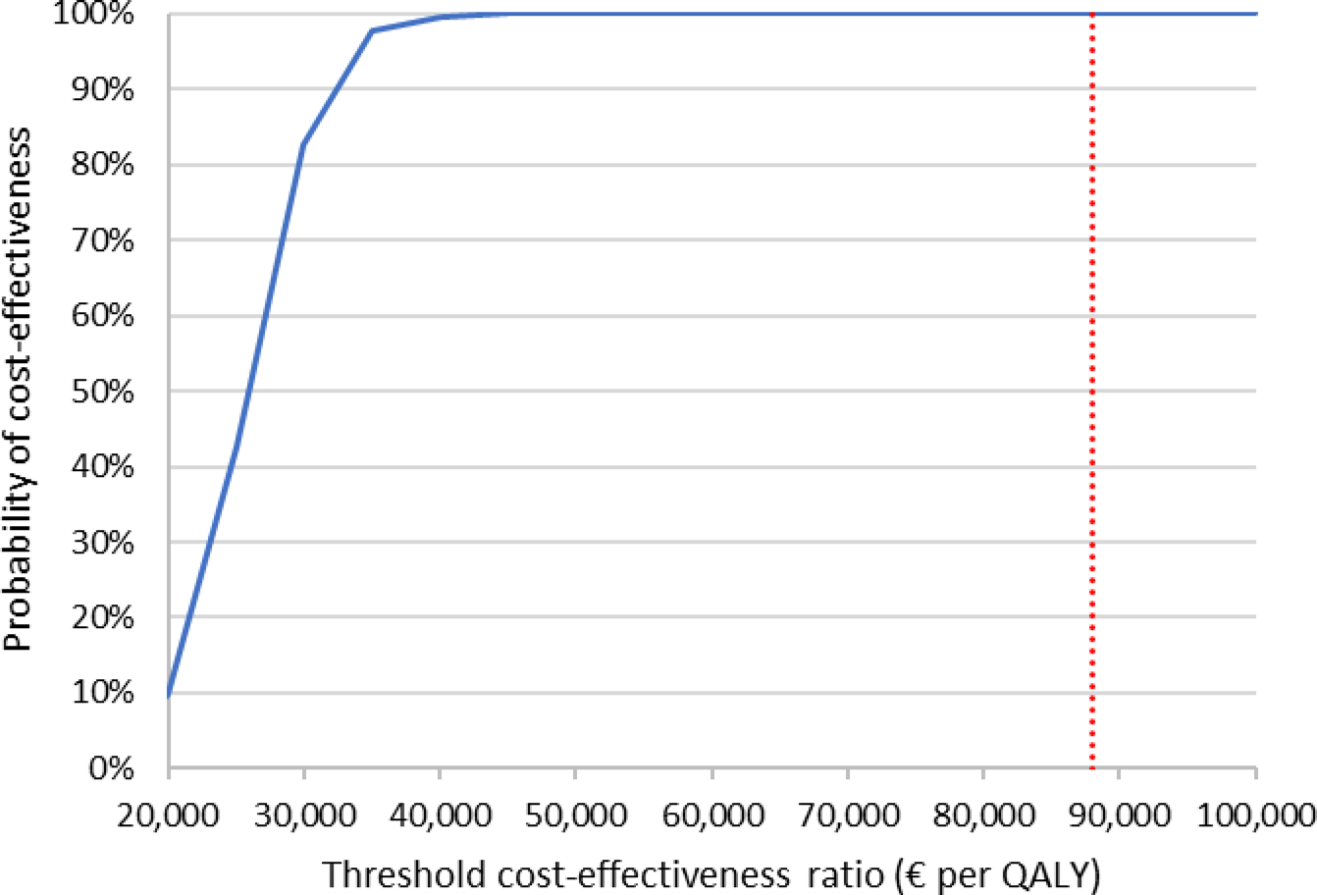
Cost-effectiveness acceptability curve (CEAC) for a six-month drug stockpiling strategy. The dashed red line indicates the base-case willingness-to-pay (WTP) threshold of €88,000 per QALY gained, representing the opportunity cost benchmark used to determine cost-effectiveness. QALY, quality-adjusted life year.

The PSA yielded a CEAC (Fig. 8), which illustrates the probability that the intervention (i.e., a six-month drug stockpiling period) is cost-effective at different WTP thresholds. At the base-case threshold of €88,000 per QALY, the probability of cost-effectiveness was 100%. Starting from around 10% at €20,000 per QALY, the probability rises steeply between approximately €20,000 and €35,000, exceeds 95% by about €35,000, and reaches 100% at roughly €40,000 per QALY, remaining at this level for higher thresholds.

## Discussion

Based on the results of the cost-effectiveness model, this study supports extending the inventory duration as proposed in the German law against supply bottlenecks for pharmaceuticals. The analysis shows that increasing the inventory duration can substantially mitigate drug shortages. Empirical findings indicate that maintaining a six-month inventory could prevent approximately 70% of drug shortages, enhancing the stability of the drug supply chain in Germany, while being cost-effective with an ICER of €17,361 per QALY gained. The PSA, summarized in the CEAC, showed that the probability of cost-effectiveness exceeds 95% at around €35,000 per QALY and reaches 100% at roughly €40,000 per QALY, remaining at 100% for higher thresholds. Thus, at the base-case WTP threshold of €88,000 per QALY, there is virtually no decision uncertainty regarding whether a six-month stockpile is cost-effective. Moreover, the maximum cost-effective supply duration increased steadily with the WTP threshold, with the base-case scenario yielding a maximum duration of approximately 922 days.

Nevertheless, important uncertainty remains regarding the magnitude of net benefit and the maximum cost-effective inventory duration. The results are sensitive to key assumptions, particularly the WTP threshold itself, storage costs, and the magnitude of clinical benefits from stockpiling. If the opportunity cost of public healthcare spending is higher—implying a substantially lower WTP threshold—or if FIFO adherence is lower or clinical benefits are overestimated, shorter inventory durations may become preferable, and the cost-effectiveness of a six-month inventory would be less clear-cut.

The study also shows the variability in the duration of drug shortages across different therapeutic categories. Given that cancer and cardiovascular diseases are the leading causes of death in Germany, the long duration of drug shortages in these two categories is particularly concerning.

The additional stockpiling costs, estimated at approximately €163 million annually in the base case, appear relatively modest compared to alternative measures such as drug price increases or reshoring pharmaceutical production to Europe or Germany. This highlights the efficiency of inventory-building strategies over these alternatives. While the storage costs may seem insignificant relative to the total annual spending by sickness funds, which exceeds €300 billion, it is still important to address how these costs might ultimately be covered.

Although the six-month stockpiling mandate legally applies to manufacturers, it remains uncertain to what extent these additional costs—for warehousing, tied-up capital, and risk of expiration—will be passed on to the SHI system. In practice, manufacturers may seek to recover these expenses through reduced rebates, higher negotiated net prices, or selective withdrawal from low-margin markets. Therefore, even if the obligation is formally imposed on manufacturers, the SHI system may still bear part or all of the cost over time. This possibility is considered in the base case, which assumes that the full economic cost of stockpiling is ultimately reflected in SHI expenditures. A more optimistic scenario, in which manufacturers absorb the costs without passing them on, would yield a more favorable ICER from the SHI perspective and strengthen the case for stockpiling even further.

One potential financing mechanism is an increase in patient co-payments. In 2021, drug co-payments amounted to €2.3 billion^27,28^. Covering the estimated €163 million in storage costs would require a 7% increase in co-payments, raising the maximum per prescription from €10 to €10.72. While this is a theoretically feasible option, its fairness and political acceptability would require further public and stakeholder debate.

While the analysis focused on clinically relevant shortages, it raises the question of whether preventing non-clinically relevant shortages may still generate value not captured in a conventional CEA, for example through option value, i.e. the value of reassurance or security associated with continued treatment availability. However, from the perspective of German SHI, inclusion of such option value is likely incompatible with the principles outlined in Book V of the Social Code (SGB V). According to § 12 SGB V, services must be sufficient, appropriate, and economical; they must not exceed what is necessary. Benefits that are deemed unnecessary or uneconomical may neither be claimed by insured persons, provided by service providers, nor approved by health insurance funds. Since option value reflects reassurance or security associated with continued treatment availability, rather than a currently necessary clinical benefit, it may fall outside the legally permissible scope of reimbursement.

This study has several limitations. First, variability in the number of reported drug shortages over time introduces uncertainty into the projections. This includes the COVID-19 pandemic period, which may have affected both shortage frequency and supply-chain dynamics. The main analysis included these years, but their influence was explored in sensitivity analyses, including an analysis that excluded the pandemic years 2020–2022 when estimating the proportion of clinically relevant shortages. Second, the assumption that manufacturers will be able to negotiate special terms or discounts with sickness funds may not fully reflect the complexities of real-world negotiations. Third, while the study accounts for the direct costs of stockpiling for Germany’s SHI system (e.g., storage, capital, and expiration), it does not quantify other relevant direct costs caused by shortages, such as increased coordination efforts, additional healthcare utilization, and follow-on treatments resulting from delayed care. From the SHI perspective, however, increased coordination costs are not directly relevant, as they are not reimbursed and are instead absorbed by pharmacies, hospitals, and physicians.

Although immediate clinical consequences such as hospitalizations or severe complications are not systematically reported in Germany, international evidence shows that such outcomes do occur and are increasingly documented. A scoping review found 11 studies that examined hospitalization status: seven reported increased hospitalizations, three reported no change, and one reported decreased hospitalizations^29^. These findings suggest that the burden of shortages on patient outcomes may be greater than previously assumed, and that at least some shortages have measurable and direct clinical consequences (e.g., emergency admissions, worsened conditions requiring inpatient care).

In the German context, the most commonly documented consequence remains treatment postponement^30,31^. While such postponements may temporarily reduce SHI spending by deferring therapy, they often lead to higher downstream costs due to disease progression, extended treatment durations, or the use of more expensive alternatives. As a result, the overall cost impact on SHI is likely underestimated because neither immediate clinical consequences nor delayed downstream costs from treatment postponement are explicitly included. Including these indirect and delayed effects would likely strengthen the case for the cost-effectiveness of the six-month stockpiling mandate and further support the implementation of an inventory reserve policy in Germany.

Fourth, given that shortage notifications are submitted by pharmaceutical manufacturers and are based on a self-imposed obligation to report supply disruptions, the publicly available BfArM database may underreport the actual number of drug shortages. Some shortages—particularly those of short duration or affecting specific pack sizes—may go unreported. However, since the CEA is restricted to clinically relevant rebated packages, which are more likely to be reported to BfArM, the impact of underreporting is likely limited. Nevertheless, to the extent that some clinically meaningful shortages are still not captured, the analysis may underestimate the health benefits of stockpiling. This would result in a conservative estimate of the cost-effective stockpiling period. In other words, the true maximum cost-effective inventory duration may be longer than the current model suggests—further reinforcing the conclusions of the study.

Fifth, the model does not explicitly account for a delay in the onset of health loss after a shortage begins. In practice, some patients may still have medication available at home for a limited period, meaning that the clinical consequences of a shortage may not arise immediately on the first day. Although the analysis is based on incremental comparisons between alternative stockpiling durations, ignoring such delays may still overestimate avoided QALY losses and thereby somewhat overstate the cost-effectiveness of stockpiling. This uncertainty is partly reflected in the sensitivity analysis using lower MCID values, which reduces the estimated QALY loss associated with shortages.

Sixth, the publicly available BfArM shortage database does not indicate whether a product is subject to a rebate contract, which limits the ability to directly stratify shortage data by rebate status. As a result, the model presented in this study reflects average conditions across all generics, irrespective of rebate status. While data from TK^32^ suggest that non-rebated generics are more likely to experience shortages on a per-product basis, rebated generics accounted for roughly 72% of all generic packages in 2023^19,20^. Because rebated generics dominate the market in volume terms, the aggregate shortage rate across all generics is nevertheless close to that observed among rebated generics.

Finally, the model is comparative-static and evaluates incremental extensions of stockpiling duration under ceteris paribus conditions. It therefore does not explicitly account for changes over time in key parameters such as shortage frequency, drug prices, or market structure. Although variation in shortage frequency was explored in sensitivity analyses, changes in drug prices or pharmaceutical market structure over time could affect the estimated cost-effectiveness of stockpiling.

The incremental cost-effectiveness of stockpiling is likely to vary across therapeutic areas, depending on factors such as drug price, shortage duration and frequency, and the expected QALY loss from treatment interruption. For example, in cancer and cardiovascular disease, shortages tend to be longer (as shown in this study), which may limit the number of shortages that can be fully prevented by a six-month reserve. However, treatment interruptions in these areas often carry more serious clinical consequences, resulting in a greater QALY loss per avoided shortage. As a result, the ICER of stockpiling in these high-severity fields may be more favorable overall, despite the lower number of preventable shortages. Conversely, in less acute conditions, where treatment interruptions have milder consequences, the value of extended reserves may be lower.

However, the goal of this analysis was not to identify the maximum cost-effective reserve duration for each drug class but to assess whether the uniform six-month stockpiling requirement, as mandated under German legislation, is economically justifiable when applied across all rebated generics. Given the absence of indication-specific reserve durations in policy, a population-weighted average approach is appropriate for assessing cost-effectiveness from the payer perspective. The model provides a conservative benchmark: if six months of inventory is cost-effective on average, it is likely even more justified for high-impact medicines. Future research could explore differentiated reserve policies by drug class or clinical priority, if sufficient granular data become available.

In response to increasing shortages of medicines, many European countries, as well as countries outside Europe, have implemented strategies that obligate manufacturers and/or wholesalers to maintain supply reserves of critically needed medicines^33^. International healthcare systems, particularly those utilizing multipayer models, can gain valuable insights from Germany’s approach to preventing drug shortages through stockpiling. In Germany, stockpiling serves as a key strategy for ensuring a stable supply of most generic medications, with a mandated duration of six months, which is notably extensive. However, the full effect of the stockpiling mandate is expected to emerge only gradually, as existing rebate contracts first need to expire before the new requirements can be fully reflected in procurement and supply patterns. Consequently, future research should focus on assessing the effectiveness of this drug inventory policy using real-world data to validate the model’s predictions.

For future policymaking, it is important to question why an inventory mandate for pharmaceutical companies should only apply to the market for rebated drugs. The German sickness fund association^34^ suggests introducing inventory requirements for manufacturers across the entire market.

## Conclusion

This study underscores the potential of a six-month inventory reserve policy in Germany to substantially mitigate drug shortages, preventing approximately 70% of supply disruptions and improving patient outcomes. The analysis demonstrates that this policy is highly likely to be cost-effective, with an ICER of €17,361 per QALY gained at a six-month storage period and a probability of cost-effectiveness approaching 100% at the opportunity-cost threshold.

While the main conclusion—that a six-month reserve is cost-effective and robust across a wide range of parameter values—is stable, important uncertainties remain regarding FIFO adherence, the health opportunity cost of public spending, and the clinical consequences of drug shortages. These uncertainties underline the need for continuous monitoring and periodic reassessment as new evidence and real-world data become available.

For policymakers, the findings emphasize the need for targeted measures to address prolonged shortages in critical therapeutic areas such as cardiovascular and oncological drugs, where the consequences of non-availability are particularly severe. Future policies could also consider extending inventory mandates beyond rebated drugs to cover a broader segment of the pharmaceutical market, thereby providing more comprehensive protection against shortages. International stakeholders may draw lessons from the German approach—especially its balance between cost-effectiveness and supply-chain resilience—as a possible template for addressing global vulnerabilities in medicine supply.

## Electronic supplementary material

Below is the link to the electronic supplementary material.

Supplementary Information.

## Data Availability

The datasets analysed during the current study are available from the BfArM repository via the PharmNet.Bund public portal [https://anwendungen.pharmnet-bund.de/lieferengpassmeldungen/faces/public/meldungen.xhtml].
