# Supplementary material for: The cost-effectiveness of inventory reserves for preventing drug shortages in Germany: a health-economic evaluation
Source: Sci Rep. 2026 Jun 8;16:17699. doi: 10.1038/s41598-026-53010-8 (PMC13247039; doi:10.1038/s41598-026-53010-8)
Supplement: Supplementary file 1 — Supplementary Information. [file 41598_2026_53010_MOESM1_ESM.docx]

Appendix. Mathematical model

To determine the maximum cost-effective storage period under a cost-per-QALY threshold (*λ*), a logarithmic function was fitted to the data describing the relationship between the quantity of avoided clinically relevant drug shortages and the number of storage days:

$y(x)=a\ln x$, $\left( 2 \right)$

where $y$ is the quantity of avoided clinically relevant drug shortages (in packs) over one year, $x$ is the number of storage days, and $a$ is a coefficient. The derivative of $y$ measures the instantaneous rate of change in the quantity of avoided relevant drug shortages with respect to the number of storage days:

$y^{'}\left( x \right)=\frac{a}{x}.$ $\left( 3 \right)$

Avoided shortages were then translated into health gains by multiplying the number of avoided package shortages by the QALY gain per avoided package, denoted by $q$. Thus,

$$\begin{matrix} & h(x)=q\text{ }y(x), & & \text{(4)} \end{matrix}$$

where $h(x)$ is the health gain in QALYs and $q$ is the QALY gain per avoided package shortage.

Substituting Eq. (2) into Eq. (4) yields:

$$\begin{matrix} & h(x)=q\text{ }a\ln(x). & & \text{(5)} \end{matrix}$$

Differentiating with respect to $x$ gives the marginal health gain:

$$\begin{matrix} & h^{'}(x)=\frac{qa}{x}. & & \text{(6)} \end{matrix}$$

Let $C(x)$ denote total cost. The maximum cost-effective storage duration is determined by equating the marginal cost per QALY gained to the threshold $\lambda$:

$$\begin{matrix} & \lambda=\frac{C^{'}(x)}{h^{'}(x)}. & & \text{(7)} \end{matrix}$$

If the marginal cost of one additional storage day is constant, so that

$$C^{'}(x)=k,$$

then substituting Eq. (6) into Eq. (7) gives:

$$\begin{matrix} & \lambda=\frac{k}{qa/x}=\frac{kx}{qa}. & & \text{(8)} \end{matrix}$$

Solving for $x$yields the maximum cost-effective storage period:

$$\begin{matrix} & x^{*}=\frac{\lambda qa}{k}. & & \text{(9)} \end{matrix}$$

To calculate the incremental cost-effectiveness ratio (ICER) for extending the storage period from $x$ to $x+\Delta x$, we use:

$$\begin{matrix} & ICER(x\to x+\Delta x)=\frac{C(x+\Delta x)-C(x)}{h(x+\Delta x)-h(x)}. & & \text{(10)} \end{matrix}$$

For small $\Delta x$, this ICER approaches the marginal ratio in Eq. (7):

$$\begin{matrix} & ICER\approx\frac{C^{'}(x)}{h^{'}(x)}=\frac{k}{qa/x}=\frac{kx}{qa}. & & \text{(11)} \end{matrix}$$
